# Naturally rare versus newly rare: demographic inferences on two timescales inform conservation of Galápagos giant tortoises

**DOI:** 10.1002/ece3.1388

**Published:** 2015-01-13

**Authors:** Ryan C Garrick, Brittney Kajdacsi, Michael A Russello, Edgar Benavides, Chaz Hyseni, James P Gibbs, Washington Tapia, Adalgisa Caccone

**Affiliations:** 1 Department of Biology, University of MississippiOxford, Mississippi, 38677; 2 Department of Ecology and Evolutionary Biology, Yale UniversityNew Haven, Connecticut, 06520; 3 Department of Biology, University of British ColumbiaOkanagan Campus, Kelowna, British Columbia, V1V 1V7, Canada; 4 College of Environmental Science and Forestry, State University of New YorkSyracuse, New York, 13210; 5 Department of Applied Research, Galápagos National Park ServicePuerto Ayora, Galápagos, Ecuador; 6 Biodiver S.A. ConsultoresKm 5 Vía a Baltra, Isla Santa Cruz, Galápagos, Ecuador

**Keywords:** Conservation, demographic history, Galápagos giant tortoise, genetic diversity, population size

## Abstract

Long-term population history can influence the genetic effects of recent bottlenecks. Therefore, for threatened or endangered species, an understanding of the past is relevant when formulating conservation strategies. Levels of variation at neutral markers have been useful for estimating local effective population sizes (*N*_*e*_) and inferring whether population sizes increased or decreased over time. Furthermore, analyses of genotypic, allelic frequency, and phylogenetic information can potentially be used to separate historical from recent demographic changes. For 15 populations of Galápagos giant tortoises (*Chelonoidis* sp.), we used 12 microsatellite loci and DNA sequences from the mitochondrial control region and a nuclear intron, to reconstruct demographic history on shallow (past ∽100 generations, ∽2500 years) and deep (pre-Holocene, >10 thousand years ago) timescales. At the deep timescale, three populations showed strong signals of growth, but with different magnitudes and timing, indicating different underlying causes. Furthermore, estimated historical *N*_*e*_ of populations across the archipelago showed no correlation with island age or size, underscoring the complexity of predicting demographic history *a priori*. At the shallow timescale, all populations carried some signature of a genetic bottleneck, and for 12 populations, point estimates of contemporary *N*_*e*_ were very small (i.e., < 50). On the basis of the comparison of these genetic estimates with published census size data, *N*_*e*_ generally represented ∽0.16 of the census size. However, the variance in this ratio across populations was considerable. Overall, our data suggest that idiosyncratic and geographically localized forces shaped the demographic history of tortoise populations. Furthermore, from a conservation perspective, the separation of demographic events occurring on shallow versus deep timescales permits the identification of naturally rare versus newly rare populations; this distinction should facilitate prioritization of management action.

## Introduction

Many threatened or endangered species have experienced recent, severe, and sustained reductions in population size, often causing genetic bottlenecks. In these cases, the most concerning genetic outcomes include inbreeding depression, a process whereby increased homozygosity at diploid autosomal loci leads to expression of deleterious recessive alleles causing lowered individual and population fitness, and loss of genetic variation leading to reduced capacity for adaptation to changing environments (Gilpin and Soulé 1986; Cornuet and Luikart 1996). Early empirical conservation genetics studies demonstrated that levels of variation at neutral markers may be useful predictors of extinction risk of small populations in the wild (e.g., Pemberton et al. 1988; Coltman et al. 1999; Slate et al. 2000). Today, information from neutral markers is routinely integrated into management plans and used to prioritize the investment of limited conservation resources in recovery and restoration efforts (e.g., Henry et al. 2009; Sunnucks 2011; Milinkovitch et al. 2013).

A longer-term view of demographic change over time offered by evolutionary population genetics has also provided important management-relevant insights for biodiversity conservation. One of these insights has been that although genetic bottlenecks are typically considered detrimental, this classic view may be overly simplistic (Milot et al. 2007). For example, under specific circumstances, past size reductions can decrease or even eliminate the genetic load carried by a population (i.e., accumulated deleterious recessive alleles that trigger inbreeding depression; Swindell and Bouzat 2006a,b; Facon et al. 2011). Thus, understanding the historical demographic context in which a recent bottleneck occurred is important. Indeed, a long-term demographic perspective may reveal why some species – such those that have maintained small population sizes for thousands of years – may be able to persist in the face of more recent, human-mediated declines (Garnett and Zander 2011).

From a conservation perspective, there is considerable value in making a distinction between a recently bottlenecked population that was previously large and stable, versus one that has always been small or had a long history of repeated size reductions. Fortunately, different types of genetic marker data can yield insights about demography over different temporal scales. For example, analyses of diploid nuclear genotypes are often most informative on generation-to-generation (“ecological”) timescales. This is because genotypes are reshuffled each generation via gametic recombination in sexually reproducing species; population allele frequencies may also be quite labile in genetically small populations owing to the effects of drift. Conversely, analyses of gene genealogies (i.e., markers that yield information on relationships among alleles) rely on mutational processes that typically operate more slowly and therefore have the ability to generate inferences on deeper (“evolutionary”) timescales (Crandall et al. 2000; Sunnucks 2000; Garrick and Sunnucks 2006; Bohonak and Vandergast 2011). Thus, in principle, it should be possible to separate time slices of demographic history (Garrick et al. 2010a; Epps et al. 2013).

A series of analytical developments have improved our ability to understand the historical context of recent population bottlenecks. Whereas previous methods relied primarily on nuclear genotypic data (particularly fast-evolving microsatellite markers; Cornuet and Luikart 1996; Garza and Williamson 2001), coalescent approaches now leverage information on evolutionary relationships among alleles (e.g., that contained in DNA sequence data; Kuhner et al. 1998; Heled and Drummond 2008). Accordingly, inferences are no longer confined to short, generation-to-generation ecological timescales. Because genealogical data extend the temporal depths over which genetic signatures of past size changes can be identified, they can provide insights into demographic changes that pre-date direct anthropogenic impacts (Jackson et al. 2013; Palsbøll et al. 2013; but see Hoffman et al. 2011). It follows, then, that when the types of genetic data necessary for these different (albeit complementary) analyses are available, changes in population size can be investigated on contrasting timescales. Here, we refer to these as *shallow* (i.e., past ∽100 generations, i.e., ∽2500 years for Galápagos giant tortoises) and *deep* (i.e., pre-Holocene; > 10 thousand years ago, KYA) timescales. Despite the potential for some temporal overlap, this distinction serves as a useful conceptual framework.

**Figure 1:**
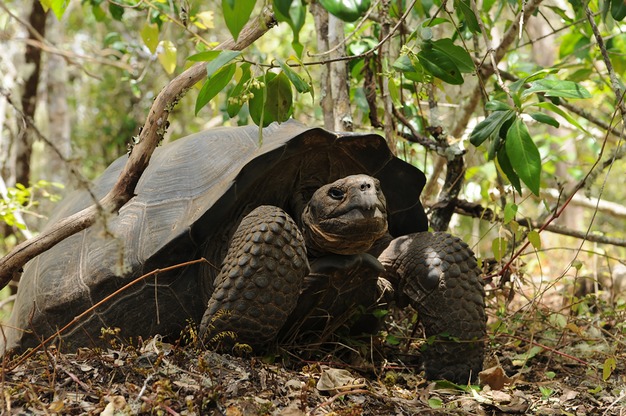
Galápagos giant tortoise (*Chelonoidis becki*) from Volcano Wolf, Isabela Island. This group represents in icon for the plight of threatened and endangered species. Genetic approaches have been extensively used to guide conservation decisions and assess management outcomes. Photo credit: Yale University.

Giant tortoises (*Chelonoidis* sp.; Fig.1) are flagships for endangered species conservation in the Galápagos Islands, particularly in the context of recent and severe population declines. When humans first arrived in the archipelago, tortoises were broadly distributed and extremely abundant (Porter 1815; Van Denburgh 1914). However, populations were decimated by buccaneers in the late 1600s and 1700s, and then by whalers and the crews of naval vessels from the late 1700s to the early 1900s (Townsend 1925; Slevin 1959). Up to 200,000 tortoises were killed within only two centuries of harvesting (MacFarland et al. 1974). Most recently, introduced mammals have become the main threats to tortoise populations, compounding the impacts of already severely reduced numbers (Cayot et al. 1994).

At present, of the 10 surviving species of Galápagos giant tortoises, four are considered vulnerable, four to five are endangered (IUCN 2014), and for some taxa such as the critically endangered *C. hoodensis* from Española Island, the situation is even worse. This species was reduced to 15 breeders four decades ago, and although more than a thousand offspring have now been repatriated from an *ex situ* breeding and rearing program, genetic diversity is still very low (Milinkovitch et al. 2004, 2013). In addition, recent work suggests that *C. elephantopus* from Floreana Island and *C. abingdoni* from Pinta Island are now represented in the wild by just a handful of individuals with mixed ancestry currently living on Volcano Wolf, Isabela Island (Russello et al. 2007, 2010; Poulakakis et al. 2008; Garrick et al. 2012; Edwards et al. 2013). Furthermore, an as-yet undescribed species from Cerro Fatal on Santa Cruz Island numbers fewer than 200 individuals, with lower genetic diversity than other species (Beheregaray et al. 2003a; Russello et al. 2005; Chiari et al. 2009).

Overlaid on the archipelago-wide negative impacts on tortoise population sizes of human activities within the past few centuries, we identify at least two other factors operating over deeper timescales that are likely to have had strong, localized effects on demography. First, Galápagos giant tortoises are a classic example of a rapid evolutionary radiation, and assuming founder-effect speciation (see Templeton 2008), the number, and genetic composition of founders that gave rise to each extant lineage is likely to have been largely stochastic. Thus, standing levels of genetic diversity in each present-day population probably reflect a combination of historical (founder effect) and contemporary (bottleneck) processes. Second, while island size alone may yield predictable demographic outcomes in some Galápagos fauna (Petren et al. 2005; Parent et al. 2008), this is confounded by within-island events. For example, approximately 100 KYA, a catastrophic volcanic eruption occurred midway along the largest and youngest island, Isabela (Geist et al. 1994). This decimated the local endemic tortoise species, *C. vandenburghi*, and the area was subsequently repopulated by a few survivors from a neighboring region (Beheregaray et al. 2003b). For these reasons, the demographic history of any given Galápagos giant tortoise population may be difficult to predict *a priori*.

**Figure 2:**
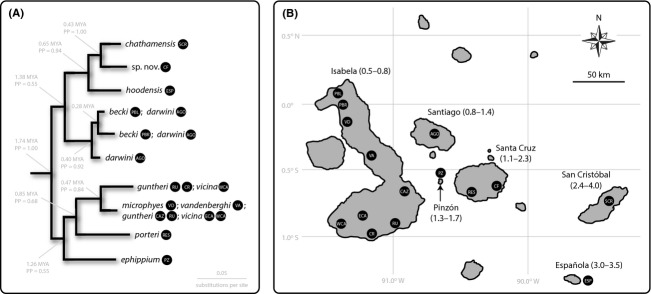
(A) Phylogenetic relationships among Galápagos giant tortoise species used in this study. The tree, including estimated node ages (in millions of years, MYA) and Bayesian posterior probability support values (PP), is simplified from Poulakakis et al. (2012). Tips of the tree are labeled with taxon names, and abbreviations in black circles are the 15 population units used for demographic analyses: PBL, Piedras Blancas; PBR, Puerto Bravo; VD, Volcano Darwin; VA, Volcano Alcedo; CAZ, Cazuela; RU, Roca Unión; CR, Cabo Rosa; ECA, eastern Cerro Azul; WCA, western Cero Azul; AGO, Santiago; PZ, Pinzón; RES, La Reserva; CF, Cerro Fatal; ESP, Española; and SCR, San Cristóbal. (B) Map of Galápagos Archipelago showing major islands, named and labeled with approximate age in million years (see Poulakakis et al. 2012 and references therein). Locations of the 15 populations are indicated by black circles, with abbreviation as above.

**Figure 3:**
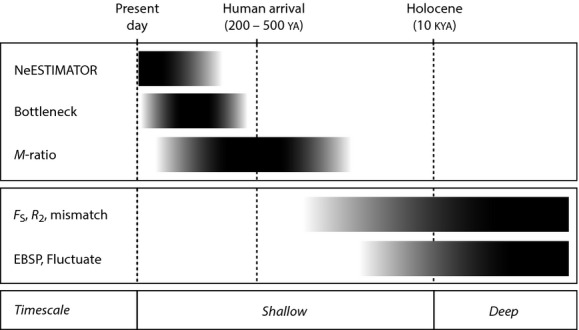
Conceptual analytical framework for reconstructing demographic history of Galápagos giant tortoises on two timescales: *shallow* (i.e., past ∽100 tortoise generations, i.e., ∽2500 years) and *deep* (i.e., pre-Holocene; >10 thousand years ago [KYA]), which pre-dates human arrival in the archipelago). Analyses are broadly partitioned into those focused on diploid genotypes and population allele frequencies versus gene genealogies *sensu* Sunnucks (2000; in this study, this generally corresponds with analyses of microsatellite versus DNA sequence data). Abbreviations and citations for methods employed in this study are as follows: years ago (YA), thousand years ago (KYA), NeESTIMATOR (Do et al. 2014), BOTTLENECK (Piry et al. 1999), *M*-ratio test (Garza and Williamson 2001), *F*_S_ (Fu 1997), *R*_*2*_ (Ramos-Onsins and Rozas 2002), mismatch distributions (Rogers and Harpending 1992), extended Bayesian skyline plot analysis (EBSP, Heled and Drummond 2008), and FLUCTUATE (Kuhner et al. 1998).

Here, we reconstructed the demographic histories of 15 Galápagos giant tortoise populations representing all 10 of the surviving species (Fig.2). This was done using a set of complementary genetic markers and analyses, within a two-timescale conceptual framework (Fig.3), to detect signatures of population size changes. Specifically, we used fast-evolving microsatellite markers as the source of genotypic and allelic frequency data for analyses of recent timescales, and more slowly evolving DNA sequences from the mitochondrial control region and a nuclear intron as the source of phylogenetic information for analyses of deep timescale demographic history. For each population, analyses addressed two questions that relate to shallow timescales: (1) Is the current effective population size (*N*_*e*_) small? and (2) Does the population show the genetic hallmarks of a recent decline? To understand the deeper time historical (pre-Holocene) context of any recent human-induced bottlenecks, we used DNA sequence data to address two additional questions: (1) Was the population's *N*_*e*_ historically small? and (2) Was the population growing, declining, or stable in size prior to human arrival?

## Methods

### DNA extraction and genetic screening

This study used previously collected samples (Caccone et al. 1999, 2002; Ciofi et al. 2002, 2006; Beheregaray et al. 2003a,b, 2004; Russello et al. 2005, 2007; Poulakakis et al. 2008, 2012; Garrick et al. 2012, 2014; Edwards et al. 2013, 2014). To facilitate analyses that generate deep timescale demographic inferences, we screened DNA sequence variation at the Paired Box protein (*PAX1P1*) intron. *PAX1P1* genes are involved in early development of animals, and their introns have previously been useful for shallow-level phylogenetics and species delimitation in turtles (Spinks et al. 2012). For 286 Galápagos giant tortoises, the most polymorphic 500-bp region of *PAX1P1* was amplified and sequenced using taxon-specific primers (see Supplementary Methods). In addition, we increased by 36 individuals our previously published mitochondrial DNA control region (mt*CR*, 704-bp) and microsatellite (12 loci) database (Russello et al. 2007; Poulakakis et al. 2008, 2012; Benavides et al. 2012; Garrick et al. 2012, 2014; Edwards et al. 2013, 2014), which underpins this study. Samples sizes for each population are given in Table1.

**Table 1:** Taxonomic and population sampling of Galápagos giant tortoises, genetic diversity at DNA sequence (mt*CR* and *PAX1P1*) and 12 microsatellite (msat) loci, and comparison of census (*N*_*c*_) and effective population size (*N*_*e*_) from NeESTIMATOR analyses. Sample size is the number of individuals assayed for variation. *N*_hap_ is the number of different haplotypes detected in each population sample; *H*_d_ is haplotypic diversity, *A*_R_ is mean allelic richness, and *H*_E_ is expected heterozygosity. All *N*_*e*_ and *N*_*c*_ values relate to the previous generation, assuming a 25-year generation time. PE is a point estimate. To calculate the *N*_*e*_:*N*_*c*_ ratio, the midpoint of the range of *N*_*c*_ values reported by MacFarland et al. (1974) was used as the *N*_*c*_ point estimate. The range of the *N*_*e*_:*N*_*c*_ ratio is the most extreme upper and lower values for this ratio

Species	Population	Sample size			DNA sequence diversity				msat diversity		*N*_c_^1^	*N*_e_	*N*_e_:*N*_c_
		*PAX1P1*	mt*CR*	msat	*PAX1P1*		mt*CR*		*A*_R_	*H*_E_	1965–1972	PE (95% CI)	PE (range)
					*N*_hap_	*H*_d_	*N*_hap_	*H*_d_					
*Chelonoidis hoodensis*	ESP	15	15	15	3	0.54	1	–	2.64	0.49	20–30	15 (6, 79)	0.61 (0.20, 3.95)
*Chelonoidis chathamensis*	SCR	17	19	19	4	0.41	1	–	5.39	0.73	500–700	68 (32, 4592)	0.11 (0.05, 9.18)
*Chelonoidis porteri*	RES	22	23	25	5	0.36	7	0.82	8.25	0.75	2000–3000	78 (45, 220)	0.03 (0.02, 0.11)
*Chelonoidis* sp. nov.	CF	13	20	21	4	0.29	2	0.10	3.83	0.54	50–100	14 (8, 27)	0.19 (0.08, 0.54)
*Chelonoidis ephippium*	PZ	21	27	24	4	0.26	13	0.88	5.93	0.67	150–200	26 (17, 45)	0.15 (0.09, 0.30)
*Chelonoidis darwini*	AGO	15	21	22	4	0.41	8	0.83	7.39	0.80	500–700	14 (11, 18)	0.02 (0.02, 0.04)
*Chelonoidis microphyes*	VD	19	21	21	3	0.50	2	0.50	4.81	0.67	500–1000	38 (21, 108)	0.05 (0.02, 0.22)
*Chelonoidis vandenburghi*	VA	25	28	24	4	0.46	5	0.33	5.46	0.67	3000–5000	102 (41, ∞)	0.03 (0.01, ∞)
*Chelonoidis guntheri*	CAZ	10	22	23	1	–	2	0.37	4.96	0.63	200–300	45 (25, 146)	0.18 (0.08, 0.73)
*Chelonoidis guntheri*	RU	10	18	17	3	0.43	3	0.54	6.73	0.75	100–200	28 (18, 48)	0.37 (0.18, 0.96)
*Chelonoidis guntheri*	CR	9	22	22	3	0.52	7	0.79	6.04	0.75	J	19 (14, 28)	0.26 (0.14, 0.56)
*Chelonoidis vicina*	ECA	22	29	29	3	0.38	6	0.42	6.79	0.76	400–600	42 (31, 65)	0.17 (0.10, 0.33)
*Chelonoidis vicina*	WCA	21	25	28	5	0.60	7	0.80	5.15	0.71	J	25 (17, 41)	0.10 (0.06, 0.21)
*Chelonoidis becki*	PBL	9	18	22	1	–	2	0.21	6.46	0.76	1000–2000	20 (16, 27)	0.03 (0.02, 0.05)
*Chelonoidis becki*	PBR	15	27	29	3	0.43	1	–	5.49	0.70	J	38 (24, 77)	0.05 (0.02, 0.15)

1 *N*_c_ values are from MacFarland et al. (1974); populations that contributed to the *N*_c_ value listed on the row above are marked with “J” (joint estimate; e.g., ECA + WCA = 400 to 600).

### Sequence alignment, phase determination, and recombination assessment

DNA sequences were edited and aligned using CLC DNA WORKBENCH v5 (http://www.clcbio.com). For the nuclear intron, length-variable heterozygotes caused by a 1-bp insertion–deletion (indel) were resolved with CHAMPURU v1.0 (Flot 2007). The final *PAX1P1* alignment comprised 428-bp of unambiguous sequence. Gametic phase of segregating sites in heterozygotes was inferred using PHASE v2.1.1 (Stephens et al. 2001), run using the search settings in Garrick et al. (2010b). Evidence for intralocus recombination was assessed with GENECONV v1.81 (Sawyer 1999), using the default mismatch penalty (*g*-scale = 0). Global permutation *P*-values (1.0 × 10^4^ replicates), based on BLAST-like global scores and Bonferroni-corrected scores for multiple comparisons, were considered indicative of recombination when *P *<* *0.05.

### Population units and genetic diversity

#### Natural genetic clusters

Analytical methods used to estimate *N*_*e*_, and investigate changes over time, assume that the underlying unit is a set of individuals randomly sampled from a genetically isolated panmictic population. Therefore, it is necessary to assess evidence for violation of assumptions. If levels of recent or ongoing gene flow are found to be non-negligible, one course of action is to identify individuals of mixed ancestry or migrant origin and exclude them prior to subsequent analyses (Waples and England 2011). This necessitates a balance between eliminating these sources of error, while still retaining sufficient sample sizes to permit meaningful demographic inferences. Comparison of mt*CR* haplotypes from this study with our existing reference database is particularly informative in this respect, as each of the major islands in the Galápagos archipelago is associated with monophyletic lineages (Poulakakis et al. 2012). Accordingly, any individual with a non-native mitochondrial haplotype can be readily identified as a long-distance migrant or a descendant of one and then omitted. To perform this first filtering step, phylogenetic relationships among mt*CR* haplotypes were estimated using TCS v1.21 (Clement et al. 2000).

In the context of our previously established reference database, nuclear microsatellite genotypes represent another source of information about the ancestry of individuals. A second filtering step was performed by estimating membership coefficients (*Q*-values) of individuals in each of *K* reference clusters, using STRUCTURE v2.3.3 (Pritchard et al. 2000; search settings as in Garrick et al. 2012). This approach can be less definitive than the above comparisons of mt*CR* haplotypes because not all microsatellite alleles are island- or species-specific, owing to long generation times coupled with relatively recent divergences. Furthermore, rare alleles or missing data can reduce *Q*-values and thus give a false impression of mixed ancestry. For these reasons, we applied a relaxed exclusion criterion to microsatellite data; only those individuals with *Q *<* *0.65 in the geographic population from which they were sampled were omitted. We recognize that there still exists a potential confounding impact of past gene flow, which would not be fully accounted for simply by removing only first generation migrants and their immediate descendants. Where possible, we examined evidence for this in our data using MIGRATE v3.5.1 (Beerli and Felsenstein 2001; details below). Finally, following Busch et al. (2007), we also assessed panmixia within groups by testing for deviations from Hardy–Weinberg equilibrium (HWE) using GENEPOP v4.0 (Rousset 2008), with sequential Bonferroni correction for multiple comparisons (Holm 1979). While failure to reject the null hypothesis of HWE does not necessarily confirm panmixia, given our sample sizes (mean of 23 individuals per population; Table1), a nonsignificant *P*-value at least provides evidence for lack of strong substructure.

#### Marker polymorphism

To quantify within-population polymorphism of *PAX1P1* and mt*CR* DNA sequences, DNASP v5.10 (Librado and Rozas 2009) was used to calculate haplotypic diversity (*H*_d_). For each microsatellite locus, we calculated allelic richness (*A*_R_) with rarefaction correction (subsampling 13 diploid individuals per population) and expected heterozygosity (*H*_E_), in HP-RARE v1.0 (Kalinowski 2005). Multilocus averages summarized *A*_R_ and *H*_E_ diversity metrics per population.

### Analyses of shallow timescales (past ∽100 tortoise generations, i.e., ∽2500 years)

#### Effective population size

To estimate contemporary *N*_*e*_, microsatellite data were analyzed with NeESTIMATOR v2 (Do et al. 2014), using the linkage disequilibrium-based method. This requires sampling at only a single time point and estimates *N*_*e*_ for the previous (parental) generation. The software was run using the random-mating model, with 95% confidence limits determined by jackknifing over loci. Alleles with frequencies < 0.02 were excluded when the number of sampled individuals (*N*) was > 25. With *N *≤* *25, the criterion for excluding rare alleles (*P*_crit_) was set at the smallest value consistent with 1/2*N* < *P*_crit_ < 1/*N*. Where possible, *N*_*e*_ values were compared to temporally and spatially corresponding estimates of population census size (*N*_*c*_) from extensive habitat survey data reported by MacFarland et al. (1974). These surveys were conducted over periods ranging from 1–10 years (minimum of three surveys per population), and with the exception of hatchlings ≤ 2 months old, included individuals of all ages. In some instances, a single *N*_*c*_ estimate reported by MacFarland et al. (1974) relates to a pair of genetic populations in this study (“joint estimates” herein). In these cases, the reported *N*_*c*_ was divided equally between the two contributing genetic populations.

Following Waples and England (2011), we examined the stability of *N*_*e*_ estimates as a function of *P*_crit_, as this can be informative about past or ongoing migration and gene flow. Briefly, *N*_*e*_ estimates from isolated populations should show little variation across a range of different (albeit small) *P*_crit_ values, whereas populations that have a history of gene flow and/or possess first generation migrants contain “foreign” alleles, which can lead to considerable variation. For each tortoise population, we re-estimated *N*_*e*_ for *P*_crit_ values spanning the range 0.02 to 0.07 and plotted the point estimates and their associated confidence intervals.

#### Population decline

Evidence for recent reductions in *N*_*e*_ was assessed using microsatellite data analyzed in BOTTLENECK v1.2.02 (Piry et al. 1999). This approach contrasts loss of rare alleles with loss of heterozygosity and can provide insights into size changes that occurred on very recent timescales. For example, Peery et al. (2012) reported that after ∽10 generations since a bottleneck event occurred, this test's power to detect it becomes very low under the conditions that were examined *in silico*. More generally, Cornuet and Luikart (1996) noted that with this test, a bottleneck is likely to be detectable after initiation of decline for only 0.25–2.5 × 2*N*_*e*_ generations (where *N*_*e*_ is the bottleneck effective population size). To generate distributions of the difference between expected heterozygosity (*H*_E_) and expected heterozygosity under mutation-drift equilibrium (*H*_EQ_), we used 1000 simulation iterations (Piry et al. 1999) and examined a broad range of combinations of single- and multistep mutation models. The null hypothesis of no heterozygosity excess was assessed using Wilcoxon's sign rank test (one-tailed, 0.05 significance level).

We also analyzed microsatellite data using the *M*-ratio test (Garza and Williamson 2001), for which the window of maximum power of detection of a past bottleneck is either within the past ∽10 to 50 generations (Peery et al. 2012), or up to (perhaps beyond) ∽100 generations, depending on the size of the postreduction population (Garza and Williamson 2001). This ratio is based on the total number of alleles at a locus versus the size range of those alleles, which changes at a slower rate than expected heterozygosity. For each population, *M* and its critical value (the point at which a past bottleneck event can be inferred) were calculated using the programs M P VAL and CRITICAL M, respectively (Garza and Williamson 2001). A bottleneck event is inferred if the empirical *M* is significantly small; this was determined via comparison with a distribution of *M*-values simulated for 10,000 theoretical populations at mutation-drift equilibrium.

When performing the *M*-ratio test, user-defined parameters include Θ (4*N*_*e*_*μ* for diploid autosomal genes, where *N*_*e*_ is the prebottleneck effective population size and *μ* is the microsatellite mutation rate), *p*_*g*_ (the proportion of mutations greater than a single step), and Δ_g_ (the mean size of multistep mutations). We used *p*_*g *_= 0.2 and Δ_g_ = 3.5 following Garza and Williamson (2001) and examined a range of Θ-values corresponding to *N*_*e *_= 50, 500, 1000, and 1500. Both slow and fast microsatellite mutation rates were used in this study (i.e., *μ *= 1.5 × 10^−4^ and 1.5 × 10^−3^), which are consistent with values reported for herpetofauna and long-lived vertebrates (Zhang and Hewitt 2003). To explore whether recent population declines are coupled with mating among close relatives, for each population we also calculated the inbreeding coefficient (*F*) and observed heterozygosity (*H*_O_) using COANCESTRY v1.0.0.1 (Wang 2011) and GENEPOP, respectively (see Supplementary Methods).

### Analyses of deep timescales (pre-Holocene, >10 KYA)

#### Evaluation of the assumption of long-term population genetic isolation

This study includes three tortoise species (i.e., *C. becki*,*C. vicina,* and *C. guntheri*) for which multiple local populations exist. Accordingly, we explored evidence for past intraspecific gene flow and assessed its potential impact on estimates of *N*_*e*_, with DNA sequence data analyzed using MIGRATE. We first addressed the question of whether there has been non-negligible historical gene flow, as measured by *M* (mutation-scaled immigration rate, *m*/*μ*). This was done by comparing parameter values estimated within a full migration matrix to those estimated within a constraint matrix representing the null hypothesis of no gene flow (i.e., *M *=* *0), via a likelihood ratio test (LRT). We also addressed the question of whether the inferred *amount* of past gene flow is likely to have a non-negligible impact on estimates of historical *N*_*e*_ (where *θ *= *N*_*e*_*μ* for mt*CR*, or 4*N*_*e*_*μ* for *PAX1P1*, served as a proxy for *N*_*e*_). The null hypothesis of no meaningful effect of gene flow on estimated *N*_*e*_ (i.e., *θ*_*M* = 0 _= *θ*_*M* > 0_) was again evaluated via LRT (see Supplementary Methods).

#### Single-locus estimates of *N*_*e*_ and changes over time

To obtain estimates of the long-term harmonic mean of *N*_*e*_, and to distinguish among hallmarks of historical population growth, decline or size constancy, we analyzed DNA sequences using FLUCTUATE v1.4 (Kuhner et al. 1998). This method generates maximum-likelihood estimates of two parameters: Θ (*N*_*e*_*μ* for mtDNA or 4*N*_*e*_*μ* for diploid autosomal genes, where *μ* is the per-lineage per-generation mutation rate), and *g* (the exponential growth parameter). All runs employed the following search strategy: 10 short Monte Carlo chains (10,000 steps) and five long chains (100,000 steps) sampling every 20th step, random starting trees, empirical base frequencies, transition/transversion ratio = 2, and initial value of Θ set using Watterson's (1975) estimate. The parameter Θ was estimated jointly with *g* (initial value of *g *=* *0.1). Runs were repeated five times, and then, the mean and standard deviation (SD) of maximum-likelihood parameter estimates were calculated.

To convert Θ to *N*_*e*_, we assumed a generation time of 25 years for Galápagos giant tortoises (Throp 1975), and a substitution rate of 3.4% per lineage per million years (MY) for mt*CR* (Beheregaray et al. 2004), giving a coarse estimate of *μ *= 8.5 × 10^−7^ per lineage per generation (*N*_*e*_ was not calculated from *PAX1P1* because substitution rate estimates from Galápagos giant tortoises or closely related taxa are not available). We note, however, that generation times are likely to vary, and overlapping (cf. discrete) generations contribute an additional source of error (Waples and Yokota 2007; Waples 2010). Interpretation of *g* followed Lessa et al. (2003), where strong evidence for growth was inferred when *g* - 6 × SD(*g*) > 0 (and for decline, *g *+* *6 × SD(*g*) < 0). FLUCTUATE requires a minimum of three different haplotypes in a dataset; as this level of variation was not available for all mt*CR* and *PAX1P1* datasets (Table1), not all populations could be analyzed using this method.

The frequency distribution of DNA sequence haplotypes can carry signal of growth or decline, so we also evaluated evidence for past size changes using Fu's (1997) *F*_S_. Simulations have shown that Ramos-Onsins and Rozas' (2002) *R*_2_, a metric based on segregating site frequency distribution, is superior to *F*_S_ when sample sizes are small. Both summary statistics were estimated from mt*CR* and *PAX1P1* sequences in each population, using DNASP. Deviation from the null hypothesis of constant size was evaluated by comparing observed values against distributions simulated via neutral coalescence (2.0 × 10^3^ replicates), with significance assessed at the 0.05 level (lower tail). For comparison, we also calculated mismatch distributions (Rogers and Harpending 1992) using ARLEQUIN v3 (Excoffier et al. 2005; see Supplementary Methods).

#### Multilocus estimates of *N*_*e*_ and changes over time

Extended Bayesian skyline plot analysis (EBSP, Heled and Drummond 2008) is a multilocus framework for estimating *N*_*e*_ and assessing evidence for historical population growth or decline, using DNA sequences. This method, implemented in BEAST v1.6.1 (Drummond and Rambaut 2007), accommodates unlinked loci with different ploidy and modes of inheritance, making it suitable for joint analysis of mt*CR* and *PAX1P1*. To retain the full set of different haplotypes observed within populations and thus maximize historical signal, indels were recoded as transitions (Castro et al. 2007). Searches used the best-fit substitution model (TN93 + I+G for mt*CR* [Beheregaray et al. 2004]; HKY+I+G for *PAX1P1* determined using JMODELTEST v0.1.1 [Posada 2008]), empirical base frequencies, and a strict-clock for mt*CR* (*clock.rate*,*μ *= 8.5 × 10^−7^ as for FLUCTUATE analyses, above). The *PAX1P1 clock.rate* parameter was estimated from mt*CR* (uniform prior, 0–1.7 × 10^−7^ also with a strict-clock [i.e., at least 5 ×  slower than mitochondrial DNA; Caccone et al. 2004]); this means that the resulting units of estimated *θ*-values are based on mtDNA and are therefore directly comparable to single-locus estimates of *θ* generated by FLUCTUATE analyses of mtDNA alone. Searches used a coalescent (extended Bayesian skyline) tree prior, linear skyline model, UPGMA-generated starting trees, auto-optimized tuning, with other priors as default. Final searches were performed using 1.0 × 10^8^ MCMC generations sampling parameters every 2000th step (10% discarded as burn-in). Convergence of chains was assessed via effective sample size values for key demographic parameters (all > 200) calculated using TRACER v1.5 (Rambaut and Drummond 2008), and by comparing five independent runs.

Replicate runs were pooled, and a frequency distribution was constructed to establish a 90% credible set of values for the “demographic population size changes” parameter (Heled 2010). Within the credible set, the modal number of size changes generated the primary inference (i.e., stable = 0 vs. not stable > 0). Where evidence for deviation from size constancy did exist, the secondary inference of past growth versus decline was based on visual inspection of the EBSP curve. In five populations, EBSP analyses were not performed because only one DNA sequence locus was polymorphic (Table1).

#### Integrating deep timescale inferences

Our analyses of DNA sequence data include a combination of exploratory and model-driven methods. As these have contrasting sensitivity and power, agreement among inferences may vary (Garrick et al. 2010a). To accommodate this potential source of discordance while also maintaining a focus on strong historical inference, at the deep timescale, we (1) treated population size constancy as the *a priori* null expectation and (2) overturned this in favor of past size change *only when at least two different analyses* supported the same alternative inference (i.e., growth, or decline). Given that not all DNA sequence loci will mark the same demographic events owing to differences in mutation rates and coalescent stochasticity (Templeton 2002), we did not require corroboration of inferences by both mt*CR* and *PAX1P1*.

## Results

### Sequence alignment, phase determination, and recombination assessment

Alignment of mt*CR* haplotypes, and *PAX1P1* haplotypes, was unambiguous. *PAX1P1* multisite heterozygotes were resolved with high confidence, as indicated by posterior probabilities of >0.9 across replicate PHASE runs for all but one *C. vandenburghi* individual (posterior probability = 0.5). The low-confidence haplotype pair was retained, given that exclusion of novel heterozygotes from a population genetic dataset can bias downstream analyses (Garrick et al. 2010b). GENECONV analysis did not detect globally significant inner or outer fragments (*P *=* *0.16 and 1.00, respectively), indicating that the sequenced region of *PAX1P1* is largely free of intragenic recombination, and thus satisfies assumptions of subsequent demographic analyses.

### Population units and genetic diversity

#### Natural genetic clusters

The archipelago-wide STRUCTURE analysis identified 12 genetic clusters, with most named species represented by a single cluster (Table S1; Fig. S1). Exceptions include morphologically cryptic structure of *C. becki* from northern Isabela Island, which formed two distinct clusters, plus a lack of strong genetic structure across central and southern Isabela Island, which led to pooling of two or more species into the same cluster (Table S1). This clustering solution is consistent with previous work (Ciofi et al. 2006; Poulakakis et al. 2008; Garrick et al. 2012, 2014; Edwards et al. 2013, 2014). Overall, most tortoises had strong genetic membership in their local genotypic cluster (87% with *Q *≥* *0.80, and across all individuals the mean *Q*-value was 0.90). Recent migrants or their immediate descendants were readily identified and removed. For the majority (80%) of individuals, the decision to include or exclude was based jointly on microsatellite plus mt*CR* data.

Fifteen local populations that were free of migrant or recently admixed individuals formed the basic units of our analyses. Overall, there was no strong evidence for intrapopulation substructure: 14 of the 15 populations showed no consistent deviation from HWE across multiple loci, the exception being *C. becki* tortoises from Piedras Blancas (where two loci showed homozygote excess). However, we note that lack of deviation from HWE must be considered within the constraints of our sample sizes and associated power of the statistical tests performed (i.e., null hypotheses can fail to be rejected owing to low power alone). In the few cases where two or more local populations (or species) were collapsed into the same cluster in the STRUCTURE analysis (i.e., *C. vicina* + *C. guntheri*, and *C. microphyes* + *C. vandenburghi*, + members of a peripheral population of *C. guntheri*; Table S1), we nonetheless chose to retain them as separate units for demographic analyses for the following reasons. First, even in the absence of strong genotypic differences, formally recognized species differ on the basis of mt*CR* sequences, ecological, and/or morphological characters, suggesting independent evolutionary trajectories. Second, where conspecific local populations were retained as separate units despite being members of the same genotypic cluster (i.e., eastern and western Cerro Azul, both *C. vicina*; and Cabo Rosa and Roca Unión, both *C. guntheri*; Table S1), previous work has shown that they exhibit significant microsatellite differentiation that evolved on timescales over which mutational processes operate (i.e., *R*_ST_ > *F*_ST_; Ciofi et al. 2002) and exchange very few migrants (Ciofi et al. 2006). In these cases, oversplitting is unlikely to violate assumptions of downstream demographic analyses.

#### Marker polymorphism

The mt*CR* locus was highly polymorphic (99 substitutions plus five indels; 59 haplotypes). Compared to other introns that have been assayed for variation in Galápagos giant tortoises (Caccone et al. 2004), polymorphism at *PAX1P1* was also substantial (16 substitutions plus one indel; 22 haplotypes). Overall, the number of different haplotypes per population (*N*_hap_), and haplotypic diversity (*H*_d_), were larger for mt*CR* than for *PAX1P1* (mean *N*_hap_ = 4.5 vs. 3.3, and mean *H*_d_ = 0.44 vs. 0.37, respectively; Table1). However, the nuclear intron was polymorphic within the three populations where mt*CR* was monomorphic, and based on the above diversity metrics, *PAX1P1* was more variable than mt*CR* in approximately one-third of the populations examined. Microsatellite mean allelic richness varied widely among populations (*A*_R _= 2.64–8.25), as did expected heterozygosity (*H*_E_ = 0.49–0.80; Table1).

### Analyses of shallow timescales (past ∽100 tortoise generations, i.e., ∽2500 years)

#### Effective population size

Linkage disequilibrium-based point estimates of *N*_*e*_ for the previous generation were generally small (range: 14–102; Table1). Four of 10 named tortoise species included here (*C. hoodensis, C. ephippium, C. darwini*, and *C. microphyes*) had point estimates of *N*_*e*_ < 50. The as-yet undescribed species from Santa Cruz Island (*C*. sp. nov.; Beheregaray et al. 2003a; Russello et al. 2005; Chiari et al. 2009) also had a point estimate of *N*_*e*_ < 50. There was a strong positive correlation between MacFarland et al.'s (1974) estimate of *N*_*c*_ versus our point estimates of *N*_*e*_ from microsatellite data (*R*^2 ^= 0.77). The mean ratio of *N*_*e*_:*N*_*c*_ was 0.16; however, there was considerable among-population variance (range: 0.02–0.61; Table1).

Assessment of the stability of *N*_*e*_ estimates as a function of *P*_crit_ indicated the possibility that biases caused by past gene flow may affect inferences for three Galápagos giant tortoise populations – Cazuela (*C. guntheri*) and Volcano Alcedo (*C. vandenburghi*) on Isabela Island, and La Reserva (*C. porteri*) on Santa Cruz (Fig. S2). Interactions between estimated *N*_*e*_ and *P*_crit_ did not show the same directionality (i.e., with increasing P_crit_, *N*_*e*_ of Cazuela and La Reserva increased by 40–60%, whereas it decreased by 50% for Volcano Alcedo). Nonetheless, changes in either direction can be indicative of past or ongoing migration and gene flow (Waples and England 2011). Given these results, we consider short-timescale inferences for these three populations to be tentative.

#### Population decline

BOTTLENECK analyses detected a strong signal of recent, severe, population decline only in the Española Island and San Cristóbal Island tortoise populations, and these inferences were robust to a broad range of microsatellite mutation models (Table S2). In contrast, Garza and Williamson's (2001) *M*-ratio test identified significant bottleneck events in all 15 populations. As above, these inferences were robust, largely unaffected by choice of Θ-value (Table S2). Tortoise populations from Española Island and Cerro Fatal on Santa Cruz Island show strongest signs of inbreeding, relative to other populations examined here (Fig. S3).

### Analyses of deep timescales (pre-Holocene, >10 KYA)

#### Evaluation of the assumption of long-term population genetic isolation

MIGRATE analyses of DNA sequence data from *C. becki*,*C. vicina*, and *C. guntheri* indicated significant deviation from the null hypothesis of zero historical interpopulation gene flow for each species, and the inferred levels of nonzero gene flow likely have appreciable impacts on genetic estimates of long-term *N*_*e*_ (all LRT *P*-values ≤ 0.01; Table S3). Thus, outcomes from subsequent analyses of changes in *N*_*e*_ over time in populations of these three species may be unreliable. Notably, however, maximum-likelihood estimates of *M* parameters from the *C. vicina* full migration matrix identified the eastern Cerro Azul population as a source – not sink – of migrants (Fig. S4), suggesting that in this case, downstream analyses may be largely unaffected.

#### Single-locus estimates of *N*_*e*_ and changes over time

Mitochondrial DNA-based point estimates of long-term harmonic means of *N*_*e*_, generated by FLUCTUATE under a coalescent model allowing for size population changes, ranged from 1.7 × 10^3^ (Roca Unión) to 27.8 × 10^3^ (Volcano Alcedo; Table S4). The average standard deviation around these point estimates was considerable (±1.04 × 10^3^). With the exception of Cabo Rosa, tortoise populations from southern Isabela Island (*C. guntheri* and *C. vicina*) had the lowest *N*_*e*_ values, whereas those from the middle-aged islands (Santiago, Pinzón, and Santa Cruz; Fig.2) had larger values. Owing to insufficient polymorphism, the same estimates were not possible for populations from the oldest islands, Española and San Cristóbal. Following Lessa et al.'s (2003) interpretation of *g* values, we found strong mt*CR* evidence for past growth in five populations. Of these, the same inference was well supported by *PAX1P1* only for La Reserva tortoises (Tables2 and S5). In the case of Cerro Fatal, which lacked sufficient mt*CR* polymorphism for this analysis, the *PAX1P1* locus provided clear signal of past growth. A signature of past population decline was identified only for Roca Unión, based on mt*CR* sequence data (Tables2 and S5).

**Table 2:** Integration of inferences across loci and different classes of DNA sequence-based demographic analyses. Single-locus methods include FLUCTUATE, *F*_S_, *R*_2_, and mismatch distributions. EBSP analysis is a multilocus method. Stable population size represents the null model in analyses except for mismatch distributions (which assumes growth), and “ns” indicates no significant deviation from the null. The integrated inference is based on the criterion of cross-validation among different classes of analyses (see Methods). The symbol “?” indicates cases where the procedure to fit model mismatch distribution and observed distribution did not converge. Statistics that could not be calculated owing to insufficient polymorphism are marked by “–”

		FLUCTUATE		*F*_S_/*R*_2_		Mismatch			
Species	Population	mt*CR*	*PAX1P1*	mt*CR*	*PAX1P1*	mt*CR*	*PAX1P1*	EBSP	Integrated inference
*Chelonoidis hoodensis*	ESP	–	ns	–	ns/ns	–	?	–	Stable
*Chelonoidis chathamensis*	SCR	–	ns	–	ns/ns	–	?	–	Stable
*Chelonoidis porteri*	RES	Growth	Growth	ns/ns	ns/ns	No growth	ns	ns	Stable
*Chelonoidis* sp. nov.	CF	–	Growth	ns/ns	ns/ns	ns	ns	Growth	Growth
*Chelonoidis ephippium*	PZ	Growth	ns	Growth/ns	ns/ns	?	ns	Growth	Growth
*Chelonoidis darwini*	AGO	Growth	ns	ns/ns	ns/ns	ns	ns	ns	Stable
*Chelonoidis microphyes*	VD	–	ns	ns/ns	ns/ns	?	?	ns	Stable
*Chelonoidis vandenburghi*	VA	Growth	ns	ns/Growth	ns/ns	ns	ns	ns	Growth
*Chelonoidis guntheri*	CAZ	–	–	ns/ns	–	ns	–	–	Stable
*Chelonoidis guntheri*	RU	Decline	ns	ns/ns	ns/ns	?	ns	ns	Stable
*Chelonoidis guntheri*	CR	Growth	ns	ns/ns	ns/ns	No growth	ns	ns	Stable
*Chelonoidis vicina*	ECA	ns	ns	ns/ns	ns/ns	ns	?	ns	Stable
*Chelonoidis vicina*	WCA	ns	ns	ns/ns	ns/ns	No growth	?	ns	Stable
*Chelonoidis becki*	PBL	–	–	ns/ns	–	ns	–	–	Stable
*Chelonoidis becki*	PBR	–	ns	–	ns/ns	–	?	–	Stable

Frequency distribution-based demographic summary statistics (*F*_S_ and *R*_2_) calculated from mt*CR* sequences supported inferences of past growth of Pinzón and Volcano Alcedo populations (Tables2 and S6). However, in contrast to some outcomes from FLUCTUATE, other populations showed no significant deviation from the null expectation of constant population size, possibly reflecting the low power of these summary statistics (Ramos-Onsins and Rozas 2002). Mismatch distribution analysis detected significant deviation from the null model of demographic growth for three populations (Cabo Rosa, La Reserva, and western Cerro Azul; Tables2 and S7). These inferences were based on the mt*CR* locus alone, and the procedure to fit the model mismatch and observed distribution did not converge in nine of 25 mismatch distributions, limiting the utility of this method.

#### Multilocus estimates of *N*_*e*_ and changes over time

Estimates of *N*_*e*_ were examined via snapshots taken at three points along the continuous temporal axis of EBSP curves, each pre-dating human arrival in the Galápagos: (1) at the beginning of the Holocene (10 KYA), (2) at the height of the Last Glacial Maximum (20 KYA), and (3) at the end of the penultimate interglacial (100 KYA). Overall, *N*_*e*_ values ranged from 0.8 × 10^3^ (Cerro Fatal, 100 KYA) to 14.1 × 10^3^ (Pinzón, 10 KYA; Table S4). As for FLUCTUATE, EBSP analyses showed that the southern Isabela Island populations of *C. vicina* and *C. guntheri* (except for Cabo Rosa) had low *N*_*e*_ values, whereas the middle-aged islands had larger values (again, no estimates were possible for the two oldest islands). Little discordance in the rank-ordering of populations was seen across the three selected EBSP time points (Table S4). Furthermore, absolute values of *N*_*e*_ derived from EBSP and FLUCTUATE analyses were similar in all but one case: The Volcano Alcedo population (*C. vandenburghi*) was indicated by EBSP analysis to have had a much smaller *N*_*e*_ (3.3–3.4 × 10^3^) than suggested by FLUCTUATE (27.8 × 10^3^; Table S4).

Joint analysis of *PAX1P1* and mt*CR* sequences using EBSP analysis revealed markedly different long-term demographic histories among some Galápagos giant tortoise populations (Table2 and Fig. S5). For Cerro Fatal and Pinzón tortoises, the modal number of population size changes was one (90% credible set: 0–2), with their EBSP curves showing that these size changes were in a positive direction. The magnitude of growth in Cerro Fatal was approximately twice that of Pinzón and appears to have been initiated much more recently (i.e., 600–800 generations ago; 15–20 KYA; Fig. S5). However, absolute *N*_*e*_ was estimated to be much larger for Pinzón tortoises than in the Cerro Fatal population (Table S4). In other tortoise populations, evidence from EBSP analysis for past size changes was weak, and so constancy was inferred.

#### Integrating deep timescale inferences

By treating size constancy as the *a priori* expectation and requiring cross-validation of alternative inferences on the basis of at least two different analyses (see Methods), we determined that three tortoise populations have a pre-Holocene history of growth (Cerro Fatal, Pinzón, and Santiago), whereas no populations were historically declining (Table2). Of the 12 populations for which we failed to overturn the *a priori* expectation of size constancy, this outcome is attributable to lack of information in the genetic data from Española, San Cristóbal, Piedras Blancas, Puerto Bravo, and Cazuela. However, in Cabo Rosa, La Reserva, and western Cerro Azul, the inference of a lack of historical growth received some support from mismatch distribution analysis; here, the null hypothesis of growth was rejected in favor of size constancy or decline (Tables2 and S7).

## Discussion

### Idiosyncratic forces shaped demographic histories

Disentangling the genetic signatures of historical versus recent demographic events is challenging. However, by drawing on different types of genetic data, we gained insights into the nature and magnitude of population size changes through time. In the Galápagos, idiosyncratic demographic histories could have been influenced by several spatially localized extrinsic factors, such as volcanic eruptions, lava flows, and periodic fragmentation of some islands via Pleistocene sea-level fluctuations (Ali and Aitchison 2014; Geist et al. 2014). Additionally, changes in the dynamics of inter- and intraspecific competition could have affected demographic processes (Waters 2011). Overlaid on this, intrinsic differences in species' biology may also promote contrasting responses to archipelago-wide environmental changes such as Pleistocene climatic cycles (Sequeira et al. 2012). In Galápagos giant tortoises, for example, domed versus saddleback carapace morphotypes associate with ecological rather than evolutionary divergence (Hunter et al. 2013). Indeed, the potential complexity of demographic histories that renders them difficult to predict underscores the value of reconstructions that molecular data can provide.

#### Deep timescales (pre-Holocene, >10 KYA)

Previous work on Galápagos giant tortoises has provided indications of disparate demographic histories (e.g., Beheregaray et al. 2003a,b, 2004; Ciofi et al. 2006). The present work extends upon these studies by integrating multilocus DNA sequence and microsatellite datasets, explicitly employing a two-timescale analytical framework, and by comparing inferences for 15 populations spanning the entire archipelago. Based on mt*CR* and *PAX1P1* sequence data and analyses using coalescent and haplotype frequency distribution methods, we found that three Galápagos giant tortoise populations showed the hallmarks of growth prior to human arrival in the region (Volcano Alcedo, Pinzón, and Cerro Fatal; Table2). Notably, these demographic histories had different underlying causes, given that these three populations represent distantly related species (*C. vandenburghi*,*C. ephippium*, and *C*. sp. nov.; Fig.2A) and inhabit different islands of contrasting size and age (Isabela is the largest and youngest; Pinzón and Santa Cruz are smallest and largest middle-aged islands, respectively; Fig.2B). Qualitatively, the magnitude and timing of growth also varied, with past size change in Cerro Fatal approximately twice the intensity and at least 4–5 times younger than the Pinzón event (Tables S5, Fig. S5; but note caveats regarding temporal precision of EBSP analyses reported by Smith et al. 2011). From a phylogeographic perspective, these results highlight the potential for pseudo-congruence (see Soltis et al. 2006) to mislead historical inference, particularly when demographic idiosyncrasy is pervasive (Garrick et al. 2008); only with detailed assessment of the nature and magnitude of inferred growth events do key differences become apparent.

Interestingly, some spatially proximate populations also showed contrasting demographic histories. For example, although the Cerro Fatal (*C*. sp. nov.) and La Reserva (*C. porteri*) populations are presently <20 km apart on the same island (Santa Cruz; Fig.1B), the former population has a history of growth (Table2), whereas the latter was stable in size (null hypothesis of past growth rejected by mismatch analysis of mt*CR* sequences, *P *=* *0.015; Table S7). This lends further support to existing evidence for within-island heterogeneity (e.g., Macías-Hernández et al. 2013). Indeed, the dynamic history of the Galápagos Islands underscores the risks of taking current conditions as predictors of population history. The archipelago's geography has changed significantly since the time tortoises' ancestors arrived (Poulakakis et al. 2012; Geist et al. 2014), and overlaid on this, marked environmental changes have also occurred. For example, up to ∽3 MYA, Galápagos conditions were warmer and continually humid, but then the climate cooled and temperature fluctuations increased (Wara et al. 2005), inducing vegetation zone shifts (Woodward 1987). More recently, sea-level changes formed temporary land bridges and altered island size and shape (Lambeck and Chappel 2001; Ali and Aitchison 2014). These factors undoubtedly affected the evolution of Galápagos biota (Fedorov et al. 2006). Our finding that demographic histories of Galápagos giant tortoises are not predictable from phylogenetic, geographic, or geological factors underscores the dynamism of this landscape setting.

#### Shallow timescales (past ∽100 tortoise generations, i.e., ∽2500 years)

The marked idiosyncrasy of Galápagos giant tortoise population demography also holds true at shallow timescales. Microsatellite analyses using the *M*-ratio test indicated that all sampled populations showed evidence of genetic bottlenecks, but conversely, only the ones from Española and San Cristóbal showed strong genetic signatures based on heterozygosity excess tests (Table S2). This incongruence between these two analytical approaches is potentially informative (e.g., Henry et al. 2009). Simulations have shown that the *M*-ratio is particularly sensitive when a bottleneck lasted several generations, the population subsequently made a recovery, and/or prebottleneck population sizes were large, whereas heterozygosity excess is more transient and most informative when a bottleneck was very recent, less severe, and/or prebottleneck population sizes were small (Williamson-Natesan 2005). Therefore, Española and San Cristóbal may have experienced bottlenecks that post-date earlier events common to all populations. Spatial variation in the intensity of harvesting by humans – where tortoises in coastal areas were most heavily decimated owing to accessibility – is well documented (MacFarland et al. 1974). Given the relatively small size and thus high coastline to interior ratio of Española and San Cristóbal, these two most recent bottlenecks likely reflect particularly severe harvesting by humans. However, with the current data, we cannot rule out the possibility that type II error underpins apparent temporal differences in bottlenecks affecting Española and San Cristóbal tortoises compared to other populations (see Peery et al. 2012).

### Conservation implications

There is a clear role for historical demographic reconstructions in applied conservation biology. For example, a formerly abundant, but recently declined (*newly rare*) population may have trait distributions that are poorly suited to the current demographic condition, whereas a *naturally rare* population may have evolved traits that are suited to consistently low densities (Lankau and Strauss 2011). Based on estimates of historical *N*_*e*_ from DNA sequence data, the pre-Holocene population size of Pinzón tortoises (*C. ephippium*; ≥ 7 × 10^3^ Table S4; Fig. S5) was among the largest in the archipelago despite the island's small present-day size (∽18 km^2^, Fig.2B) and long-term isolation (Geist et al. 2014). Notably, this population has very high mt*CR* haplotypic diversity (13 haplotypes, *H*_d _= 0.88), which we cautiously attribute to retained ancestral polymorphism resulting from large historical *N*_*e*_ (cf. being due to past gene flow, or sustained strongly female-biased sex ratios). Given that Pinzón tortoises are known to have been severely reduced in numbers following the arrival of humans (MacFarland et al. 1974; and *M*-ratio tests, Table S2), we categorize this population as *newly rare*.

The estimated historical *N*_*e*_ of Cerro Fatal tortoises (*C*. sp. nov.) from Santa Cruz Island (≤ 1.9 × 10^3^; Table S4; Fig. S5) was among the lowest of all populations. Phylogenetic reconstructions indicate that the Cerro Fatal population is quite young (∽0.43 MYA), most likely founded by migrants from San Cristóbal Island (Caccone et al. 2002; Beheregaray et al. 2003a, 2004; Poulakakis et al. 2008, 2012). In this case, founder-effect speciation – a process typically involving a severe historical bottleneck followed by demographic growth (Templeton 2008) – is a likely mode of divergence. Indeed, our inferences are consistent with a scenario of very few founders and sustained small population size, followed by some growth (Table2, Fig. S5). In recent years, Cerro Fatal tortoises continue to persist only in low numbers (*N*_*c *_= 50–100; MacFarland et al. 1974; Table2). Thus, in contrast to Pinzón, the available data suggest that the Cerro Fatal population can be considered *naturally rare*.

Consistent with documented human-induced bottlenecks that occurred in the last two centuries (MacFarland et al. 1974), analyses of microsatellite data showed that most Galápagos giant tortoise populations are characterized by small *N*_*e*_ (point estimates = 15–102; Table2). Relative to other tortoise populations examined here, Española, Cerro Fatal, and Pinzón show the strongest indications of potential inbreeding (Fig. S3). Of these, Española is currently the focus of intensive management intervention (Milinkovitch et al. 2004, 2013). Although the utility of general thresholds of minimum viable population size have been challenged (Garnett and Zander 2011), within the framework of Franklin's (1980) 50/500 rule and Frankham et al.'s (2014) upward revision, the short-term viability of up to 12 Galápagos giant tortoise populations – and the long-term viability of all 15 populations examined here – is of concern.

The average ratio of effective to census population size (*N*_*e*_:*N*_*c *_= 0.16; Table2) was similar to that determined from a meta-analysis of diverse wild species (*N*_*e*_:*N*_*c *_= 0.10–0.11; Frankham 1995), but we also found high variance among populations in the proportion of effective breeders. Interestingly, these results suggest that different dynamics may be operating in small versus large populations: ratios that are far below our observed average (i.e., those where *N*_*e*_:*N*_*c*_ ≤ 0.05) were all associated with relatively large census sizes (*N*_*c *_= 600–4000), whereas those well above average (i.e., *N*_*e*_:*N*_*c*_ ≥ 0.26) were seen only when census sizes were very small (*N*_*c*_ = 25–75; Table2). We stress that this apparent trend requires cautious interpretation given the sources of error relating to MacFarland et al.'s (1974) *N*_*c*_ estimates as well as our *N*_*e*_ estimates (see Methods). However, with these caveats in mind, we speculate that reproductive success of adult Galápagos giant tortoises may be partly dependent on overall population size whereby as population size increases, so too may variance in reproductive success.

### Limitations of demographic reconstructions from molecular data

Recent years have seen advances in the power and precision of reconstructions of historical demographic changes (Heled and Drummond 2008; Ho and Shapiro 2011), and identification of relatively recent bottlenecks or estimation of present-day *N*_*e*_ values (Do et al. 2014). To some extent, improvements can be achieved by increasing the number of loci (Carling and Brumfield 2007). However, a number of noteworthy constraints still exist (Palsbøll et al. 2013), and these can be broadly categorized as those relating to violation of key assumptions made by the analytical methods used, or inherent error in parameter estimates themselves.

Violation of the assumption that an isolated, panmictic population is the unit of analysis is potentially common. Unrecognized within-population substructure and/or the presence of recent gene flow can generate false genetic signatures that mimic bottleneck events (Peter et al. 2010; Heller et al. 2013), or mask size changes that did occur (Busch et al. 2007). Furthermore, biases caused by the remnants of historical gene flow can persist for considerable time (Waples and England 2011). In datasets from endangered species, sample sizes are usually limited, and thus, the impact of even a single unrecognized migrant or admixed individual is proportionately large. Thus, although removal of recent migrants from a dataset is warranted, it may nonetheless be insufficient. In this study, we found that removal of recent migrants and their immediate relatives seemed to minimize – but not eliminate – potential impacts on estimates of *N*_*e*_ in the previous generation (Fig. S2). Indeed, this data filtering step did not eliminate impacts on estimates of historical *N*_*e*_; MIGRATE analyses provided strong evidence for non-negligible past gene flow between or among conspecific tortoise populations included in our study (Table S3; Fig. S4). Although this method has its own limitations (e.g., the assumption of ancient divergence and no incomplete lineage sorting, which are unlikely to hold true for most Galápagos giant tortoises), it nonetheless highlights the need for cautious interpretation.

Another commonly violated assumption relates to the existence of discrete (c.f. overlapping) generations. Generally speaking, although age structure may have limited impact on estimators of long-term *N*_*e*_, contemporary estimates are likely to be more heavily influenced (Waples 2010). In the latter case, the sampling design may have an overarching effect on the nature and magnitude of resulting biases (Waples and Yokota 2007). Our sampling of tortoise populations typically includes individuals in the estimated age range of 12 to > 65 years old (Edwards et al. 2013). Younger juveniles have probably been included in some cases, but hatchlings are rarely encountered and thus are usually not sampled. Owing to the lack of relevant life history information, the impacts of overlapping generations on downstream analyses performed here remain unexplored, yet represent a potential source of error – particularly for shallow timescale estimates of *N*_*e*_.

Inherent error in parameter estimates may also affect inferences, particularly the ability to detect recent bottlenecks using microsatellite data (e.g., Busch et al. 2007; Peery et al. 2012). Furthermore, fixed parameters such as mutation model or rate may also be influential. In this study, historical *N*_*e*_ values estimated from DNA sequence data were unexpectedly high (Table S4) and differed substantially from the linkage disequilibrium-based estimates from microsatellites (Table2). Discrepancies between mitochondrial- and microsatellite-based estimates are not uncommon (Qiu et al. 2013) and at least partly relate to different quantities that are being measured (e.g., long-term harmonic mean vs. previous generation only; Crandall et al. 1999). In the context of DNA sequence data, at least two potential sources of error exist. First, time dependency of molecular rates may explain why direct estimates of mutations arising over the course of one or a few organismal generations tend to be much higher than phylogenetic estimates calibrated via known splitting times of sister species (Ho et al. 2005). If true, Beheregaray et al.'s (2004) phylogenetic estimate of the Galápagos giant tortoise mt*CR* substitution rate could be too slow for population-level inferences, translating into overestimates of historical *N*_*e*_ in this study. Second, simulations have indicated that FLUCTUATE shows systematic upward biases in estimates of *θ*, and thus estimated *N*_*e*_ (Kuhner et al. 1998). Recent work indicates that EBSP analyses may be subject to the same error (Smith et al. 2011). There is, however, little reason to believe that these potential biases would have affected the primary historical demographic inference (growth, decline, or size constancy). We must also consider the possibility that past tortoise populations attained substantially larger sizes than anticipated.

Despite these limitations, demographic insights from this study illustrate the value of a long-term evolutionary perspective, as well as an understanding of recent or ongoing population processes. For example, our results highlighted the idiosyncratic nature of historical population size changes in the Galápagos, prior to the arrival of humans. Such characterization of species' responses to past environmental change can provide critical baseline data for predicting future impacts (Cordellier and Pfenninger 2009). In some cases, we were also able to differentiate between newly versus naturally rare populations. Given that demographic history may be a useful predictor of which species are most likely to have adaptive differences among conservation units (Funk et al. 2012), this distinction may become useful for prioritizing candidates for more intensive management intervention.

## Data accessibility

Microsatellite genotypes and aligned DNA sequences: DRYAD entry doi:10.5061/dryad.7h8q2

## Supporting Information

**Appendix S1.** Supplementary methods. **Table S1.** Number and composition of natural genetic clusters of Galápagos giant tortoises determined by STRUCTURE analysis (Pritchard et al. ) of a reference database including representatives of all extant and most extinct species. **Table S2.** Tests for population bottleneck events that occurred on recent timescales, based on heterozygosity excess and *M*-ratio tests. **Table S3.** Exploration of evidence for historical population genetic isolation, and potential impacts of past gene flow on long-term *N*_*e*_ estimates, assessed using likelihood ratio tests (LRTs) calculated using MIGRATE (Beerli and Felsenstein). **Table S4.** Comparison of DNA sequence-based point estimates of effective population size (*N*_*e*_, reported in units of 10^3^) from two coalescent methods: FLUCTUATE (Kuhner et al.) and extended Bayesian skyline plot analysis (EBSP; Heled and Drummond). **Table S5.** Assessment of past changes in *N*_*e*_ within local populations based on maximum-likelihood estimates of *g*, the exponential growth parameter, calculated using FLUCTUATE (Kuhner et al.). **Table S6.** Assessment of signatures of past population size changes based on the frequency distribution of DNA sequence haplotypes, examined using DNASP (Librado and Rozas). **Table S7.** Assessment of signatures of past population size changes based on mismatch distribution analysis of DNA sequences (Rogers and Harpending), calculated using ARLEQUIN (Excoffier et al.). **Figure S1.** Inference of the best-fit number of natural genotypic clusters (*K*) based on STRUCTURE (Pritchard et al.) analyses of a “reference” microsatellite dataset comprising representatives of all extant and extinct Galapagos giant tortoise species (Garrick et al.) **Figure S2.** Stability of *N*_*e*_ estimates as a function of *P*_crit_, used to explore the possibility of biases introduced by past gene flow, implemented in NeESTIMATOR (Do et al.). **Figure S3.** Metrics of inbreeding, estimated using COANCESTRY (Wang ). **Figure S4.** Migration matrices estimated using MIGRATE (Beerli and Felsenstein), based on multilocus DNA sequence data (mt*CR* plus *PAX1P1*), for the three tortoise species for which multiple local populations exist. **Figure S5.** Extended Bayesian skyline plot analysis of changes in *N*_e_ over time, jointly estimated from *PAX1P1* and mt*CR* sequences, using BEAST (Drummond and Rambaut ).
