# Supplementary material for: Naturally rare versus newly rare: demographic inferences on two timescales inform conservation of Galápagos giant tortoises
Source: Ecol Evol. 2015 Jan 13;5(3):676–94. doi: 10.1002/ece3.1388 (PMC4328771; doi:10.1002/ece3.1388)
Supplement: Supplementary file 1 [file ece30005-0676-sd1.docx]

Garrick RC, Kajdacsi B, Russello MA, Benavides E, Hyseni C, Gibbs JP, Tapia W, Caccone A (2015) Naturally rare versus newly rare: Demographic inferences on two timescales inform conservation of Galápagos giant tortoises. *Ecology and Evolution*.

**Supplementary Methods**

*Amplification and sequencing of the Paired Box protein (PAX1P1) intron*

*PAX1P1* was initially amplified via Polymerase Chain Reaction (PCR) and a 900 base pair (bp) fragment was sequenced from five individuals using primers PAX.20F and PAX.21R (Kimball *et al.* 2009). Using this information, taxon-specific internal primers GalPAX-F (5’-TCTGTCATATTCATCCTCCTC-3’) and GalPAX-R (5’-CAAGCCACACATTTTTAAGG-3’) were designed, targeting the most polymorphic 500-bp fragment of this intron. For 286 Galápagos giant tortoises, the shorter region of *PAX1P1* was amplified in 13.5 µL reaction volumes containing 8.37 µL dH_2_O, 0.75 µL 5x Promega Go*Taq* Buffer, 0.9 µL Promega MgCl_2_ (25 mM), 1.2 µL New England Biolabs (NEB) dNTP mix (10 µM), 0.9 µL NEB bovine serum albumin (100×), 0.6 µL each primer (10 µM), 0.18 µL Promega Go*Taq* (5U/µL), and 1.5 µL genomic DNA. PCR cycling conditions were: 95°C 5 min initial denaturation (1 cycle), 95°C 30s, 50°C 30s, 72°C 1 min (35 cycles), and 72°C 5 min final extension (1 cycle). Amplicons were purified using ExoSap (NEB), and sequenced on an Applied Biosystems 3730 at Yale University’s DNA Analysis Facility on Science Hill.

**Analyses of shallow timescales (past ~100 tortoise generations, i.e., ~2500 years)**

*Inbreeding*

To determine whether mating among close relatives has been shaping present-day levels of genetic diversity, the inbreeding co-efficient (*F*) was calculated from microsatellite loci with COANCESTRY v1.0.0.1 (Wang 2011), using Lynch & Ritland’s (1999) moment method, and Wang’s (2007) triadic maximum-likelihood method. Since *F* tends to increase as the duration and/or severity of inbreeding increases whereas observed heterozygosity (*H*_O_) decreases, for comparison we also calculated *H*_O_ using GENEPOP, with *H*_O_ averaged across loci for each population.

**Analyses of deep timescales (pre-Holocene, > 10 KYA)**

*Evaluation of the assumption of long-term population genetic isolation*

For analyses of DNA sequence data using MIGRATE v3.5.1 (Beerli & Felsenstein 2001), the full migration matrix was comprised of four parameters in each of the *C. becki* and *C. vicina* two-population models (i.e., θ_1_ and θ_2_, where θ = *N_e_*µ for mt*CR*, or 4*N_e_*µ for *PAX1P1*; and *M*_1→2_ and *M*_2→1_, where *M* = migration rate/µ), or nine parameters in the three-population *C. guntheri* model (three θ-values and six *M*-values). When assessing evidence for non-negligible gene flow over time, we used a constraint matrix in which all *M*-values were fixed at a very small value (i.e., 0.1) rather than at zero, because the latter would not lead to a single coalescent tree (P. Beerli, pers. comm.). To investigate whether the inferred level of past gene flow is likely to have a non-negligible impact on estimates of historical *N_e_*, we first estimated θ (*N_e_*µ for mt*CR*, or 4*N_e_*µ for *PAX1P1*) for each population under a model of complete isolation (*M* = 0), and the resulting value was used to seed the constraint matrix of a subsequent run. In all cases, we used the following MIGRATE search settings were employed: 10 short MCMC chains (30,000 steps), three long chains (300,000 steps) recording every 100th genealogy, 30,000-genealogy burn-in per chain, MC^3^ heating (temperatures 1.0, 1.5, 2.5, and 4.0), UPGMA starting trees, empirical base frequencies and transition/transversion ratio of 2.0. Initial values for θ and *M* were set using *F*_ST_. All parameter estimates were generated by combining five replicate runs. DNA sequences were analyzed as both single- and multilocus datasets; in the latter case, an inheritance scaler of 1:4 (mt*CR* : *PAX1P1*) was used.

*Single locus estimates of N_e_* *and changes over time*

In contrast to *F*_S_ and *R*_2_, analyses of the distribution of the pairwise sequence differences (mismatch distributions) assume demographic growth (Rogers & Harpending 1992). This alternative null hypothesis provides an opportunity to assess strength of evidence for long-term stability in population size, which is typically characterized by a multimodal, ragged, mismatch distribution. We used ARLEQUIN v3 (Excoffier *et al.* 2005) to compute mismatch distributions for each locus and population, and used the generalized least-squares approach (Schneider & Excoffier 1999) to test the empirical mismatch distributions for significant deviation from a model of demographic growth (10,000 permutations).

**Supplementary Tables**

**Table S1.** Number and composition of natural genetic clusters of Galápagos giant tortoises determined by STRUCTURE analysis (Pritchard *et al*. 2000) of a reference database including representatives of all extant and most extinct species. Population abbreviations follow Fig. 1 of the main text. Twelve clusters were recovered. Most named species form a single cluster, although some geographically neighboring species from Isabela Island clustered together (07 and 08), while two populations of the same species (*C. becki*) from Volcano Wolf on Isabela Island were split into two clusters (11 and 12). *N* is the number of purebred individuals per cluster included in the reference database (from Garrick *et al*. 2012)


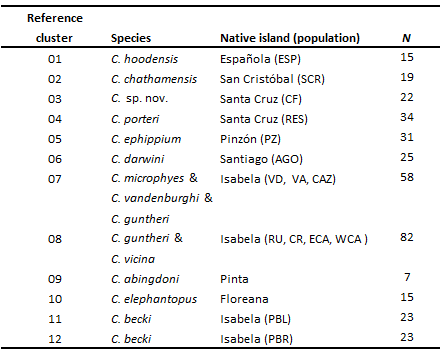


**Table S2.** Tests for population bottleneck events that occurred on recent timescales, based on heterozygosity excess and *M*-ratio tests. Population abbreviations follow Fig. 1 of the main text. Heterozygosity excess tests were implemented in BOTTLENECK (Piry *et al.* 1999), assuming different microsatellite mutation models (SMM = strictly a single-step mutation model; TPM = two-phase mutation model, with the numeric suffix indicating the proportion of mutations that do follow single-step). *M*-ratio tests were implemented in the M P VAL (Garza & Williamson 2001), assuming different values of theta (Θ = 4*N_e_μ*) and using a two-phase mutation model where 80% of mutations are single-step and the mean multi-step size = 3.5 repeats. For both tests, *P*-values are reported, with significance levels indicated as follows: *** < 0.001, ** < 0.01, * < 0.05, ns = not significant.


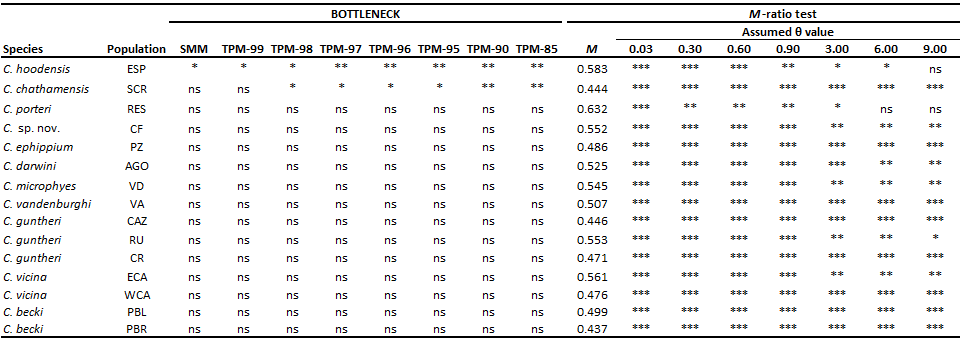


**Table S3.** Exploration of evidence for historical population genetic isolation, and potential impacts of past gene flow on long-term *N_e_* estimates, assessed using likelihood ratio tests (LRTs) calculated using MIGRATE (Beerli & Felsenstein 2001). Two null hypotheses were considered: (1) zero migration between conspecific populations (*M* = 0), and (2) no impact of any past migration on estimates of the product of *N_e_* and µ (θ*_M_* _= 0_ = θ*_M_* _> 0_). The table reports *P*-values for tests based on single- and multilocus datasets. Population abbreviations follow Fig. 1 of the main text.


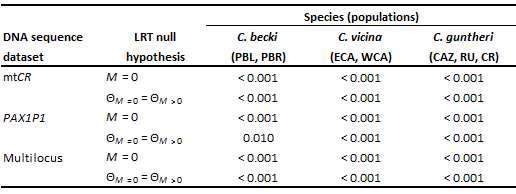


**Table S4.** Comparison of DNA sequence-based point estimates of effective population size (*N_e_*, reported in units of 10^3^) from two coalescent methods: FLUCTUATE (Kuhner *et al*. 1998) and extended Bayesian skyline plot analysis (EBSP; Heled & Drummond 2008). Population abbreviations follow Fig. 1 of the main text. FLUCTUATE provides a single-locus (mt*CR*) estimate that represents a long-term harmonic mean. EBSP provides a multilocus estimate (mt*CR* plus *PAX1P1*) that was examined at three points that pre-date human arrival in the Galápagos (reported in thousands of years ago, KYA; also see Fig. S4). All reported *N_e_* values were averaged across independent replicate runs. Color codes indicate rank-ordering of populations, from large to small *N_e_* (i.e., ‘hot’ dark red to ‘cool’ dark blue, respectively). Statistics that could not be calculated owing to insufficient polymorphism are marked by “–”.


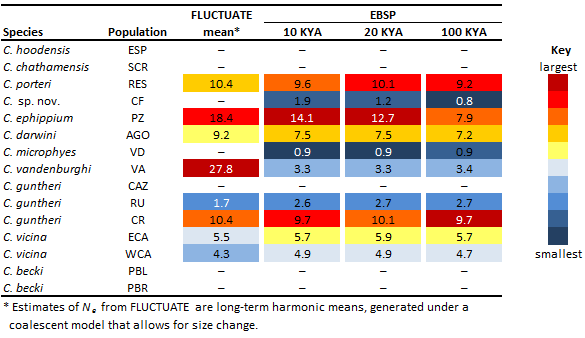


**Table S5.** Assessment of past changes in *N_e_* within local populations based on maximum-likelihood estimates of *g*, the exponential growth parameter, calculated using FLUCTUATE (Kuhner *et al*. 1998). Population abbreviations follow Fig. 1 of the main text. The significance of *g* was interpreted following Lessa *et al*. (2003), where large positive values indicate growth and negative values indicate decline. Mean and standard deviation (SD) of *g* were calculated from five replicate runs per locus per population. Statistics that could not be calculated owing to insufficient polymorphism are marked by “–”.


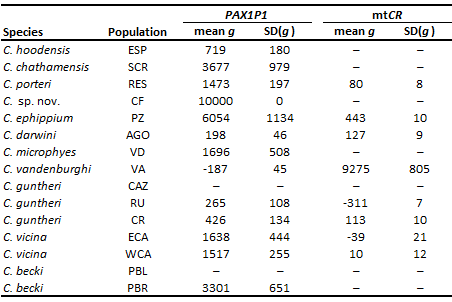


**Table S6.** Assessment of signatures of past population size changes based on the frequency distribution of DNA sequence haplotypes, examined using DNASP (Librado & Rozas 2009). Summary statistics *F*_S_ (Fu 1997) and *R*_2_ (Ramos-Onsins & Rozas 2002) were calculated for each polymorphic locus. Population abbreviations follow Fig. 1 of the main text. Deviation from the null hypothesis of size constancy was assessed using coalescent simulations. Significantly small *R*_2_ (marked by †) or negative *F*_S_ indicates growth, whereas significantly large *R*_2_ or positive *F*_S_ indicates decline (* represents *P* < 0.05). Statistics that could not be calculated owing to insufficient polymorphism are marked by “–”.


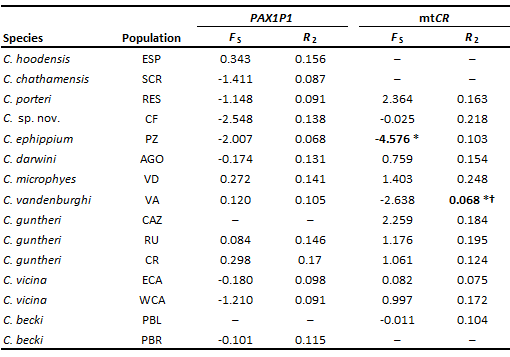


**Table S7.** Assessment of signatures of past population size changes based on mismatch distribution analysis of DNA sequences (Rogers & Harpending 1992), calculated using ARLEQUIN (Excoffier *et al.* 2005). Population abbreviations follow Fig. 1 of the main text. Deviation of the empirical data from a null model of demographic growth was assessed via permutation using the generalized least-squares approach (Schneider & Excoffier 1999), with significance assessed at the 0.05-level. Parameters of the model are as follows: τ, relative time since population expansion; θ_0_ and θ_1_ are relative population sizes before and after expansion, respectively. The symbol “?” indicates those cases where the procedure to fit the model mismatch and observed distribution did not converge. Statistics that could not be calculated owing to insufficient polymorphism are marked by “–”.


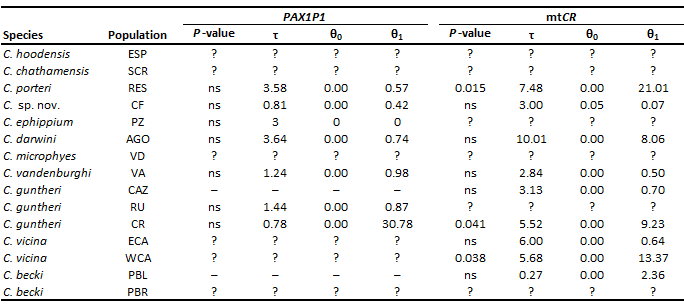


**Supplementary Figures**


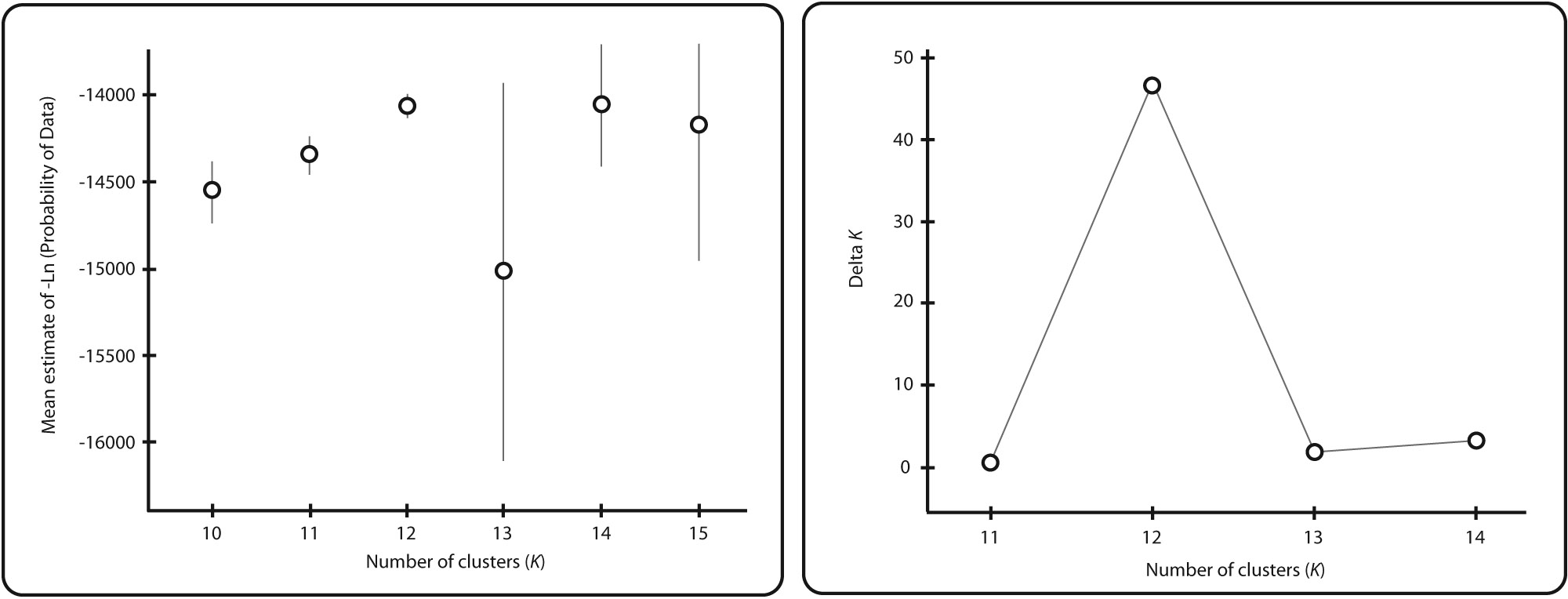


**Fig. S1.** Inference of the best-fit number of natural genotypic clusters (*K*) based on STRUCTURE (Pritchard *et al*. 2000) analyses of a ‘reference’ microsatellite dataset comprising representatives of all extant and extinct Galapagos giant tortoise species (Garrick *et al*. 2012). The left graph shows the choice of *K* = 12 based on the relationship between the estimated log likelihood of the data and increasing *K*. Following Pritchard *et al*. (2000), the smallest value of *K* that captured the major structure in the data was taken as ‘correct’. The right graph shows further confirmation of choice of *K* = 12, based on the second order rate of change of the likelihood function (Δ*K*; Evanno *et al*. 2005).

**
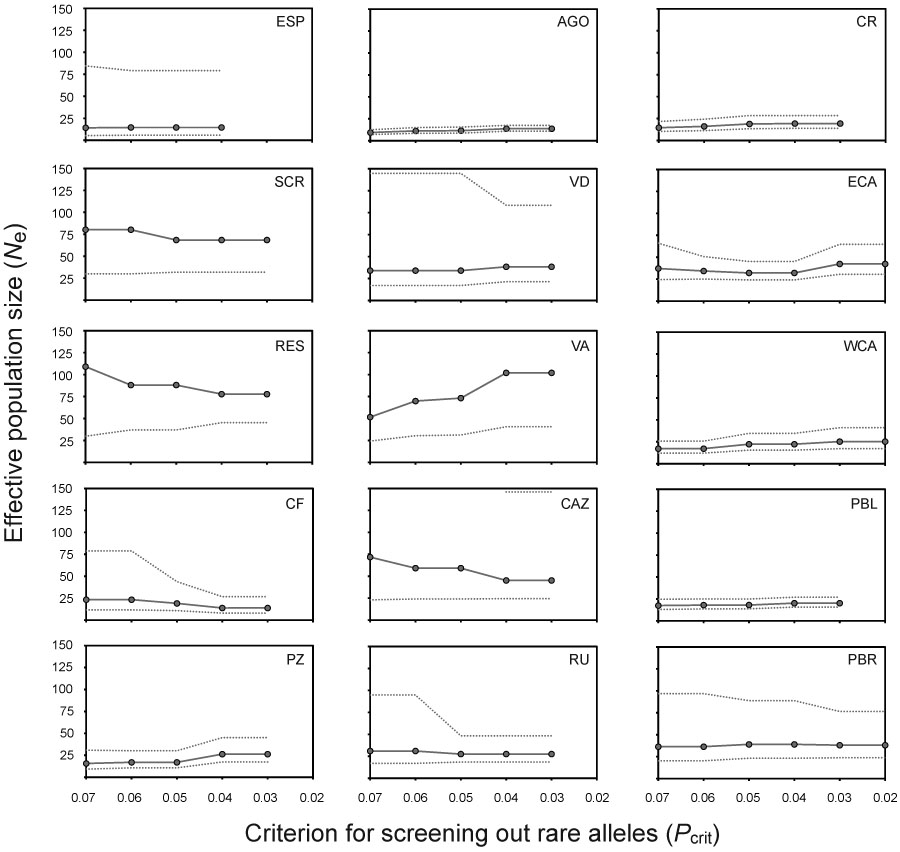
**

**Fig. S2.** Stability of *N_e_* estimates as a function of *P*_crit_, used to explore the possibility of biases introduced by past gene flow, implemented in NeESTIMATOR (Do *et al*. 2014). Each panel represents a different tortoise population (abbreviations follow Fig. 1 of the main text), the solid line represents the point estimate of *N_e_*, and dashed lines indicate associated confidence intervals. *N_e_* was not calculated for *P*_crit_ values that are too low to screen out alleles that occur as only a single copy among the sampled individuals (see Table 1 of the main text for population sample sizes).


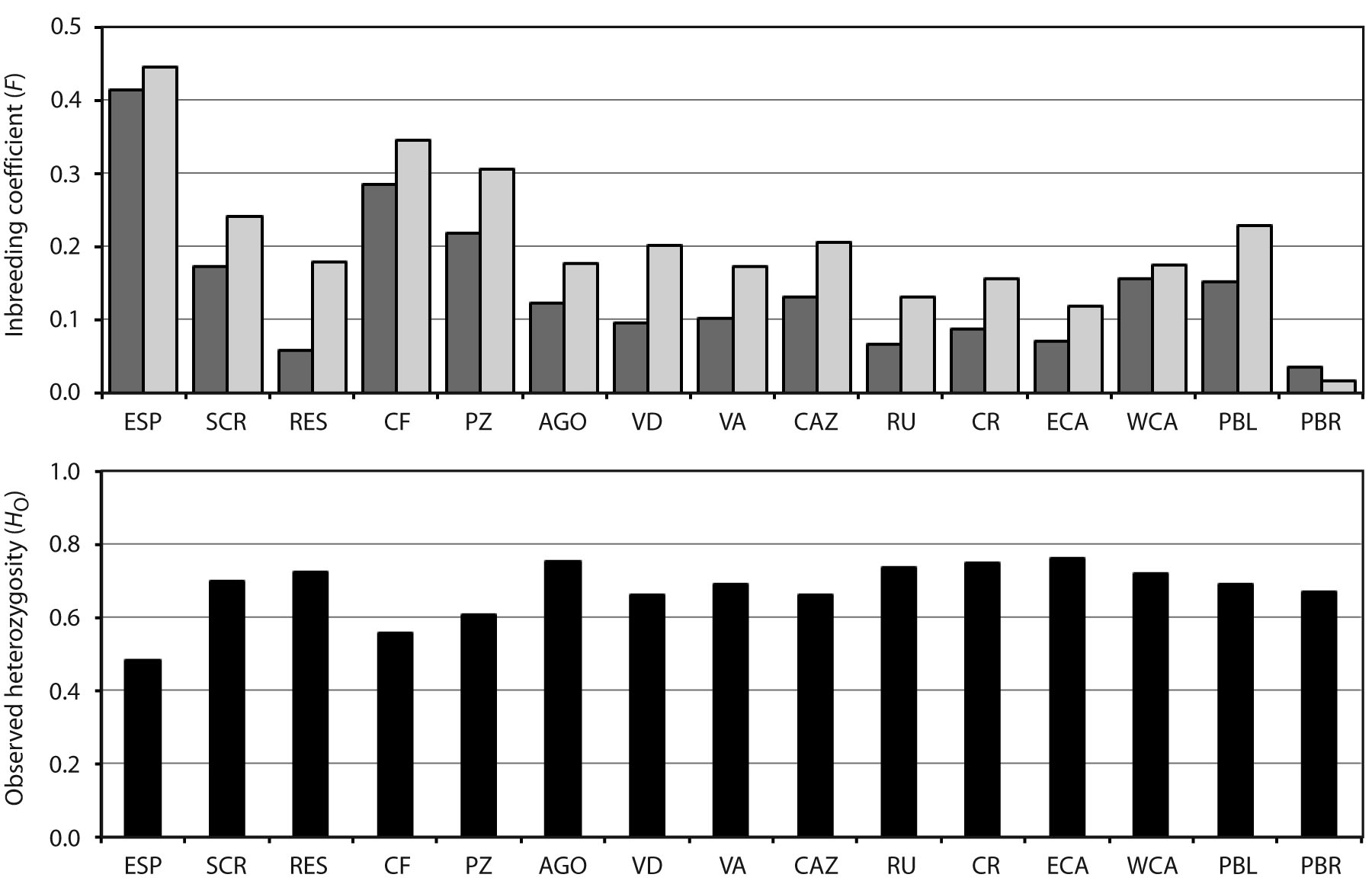


**Fig. S3.** Metrics of inbreeding, estimated using COANCESTRY (Wang 2011). Top panel: mean inbreeding coefficient (*F*) estimated using Lynch and Ritland’s (1999) moment method (dark grey bars), and Wang’s (2007) triadic maximum-likelihood method (pale grey bars). Lower panel: observed heterozygosity (*H*_O_) averaged across 12 microsatellite loci (black bars). Population abbreviations (*x*-axis) follow Fig. 1 of the main text.


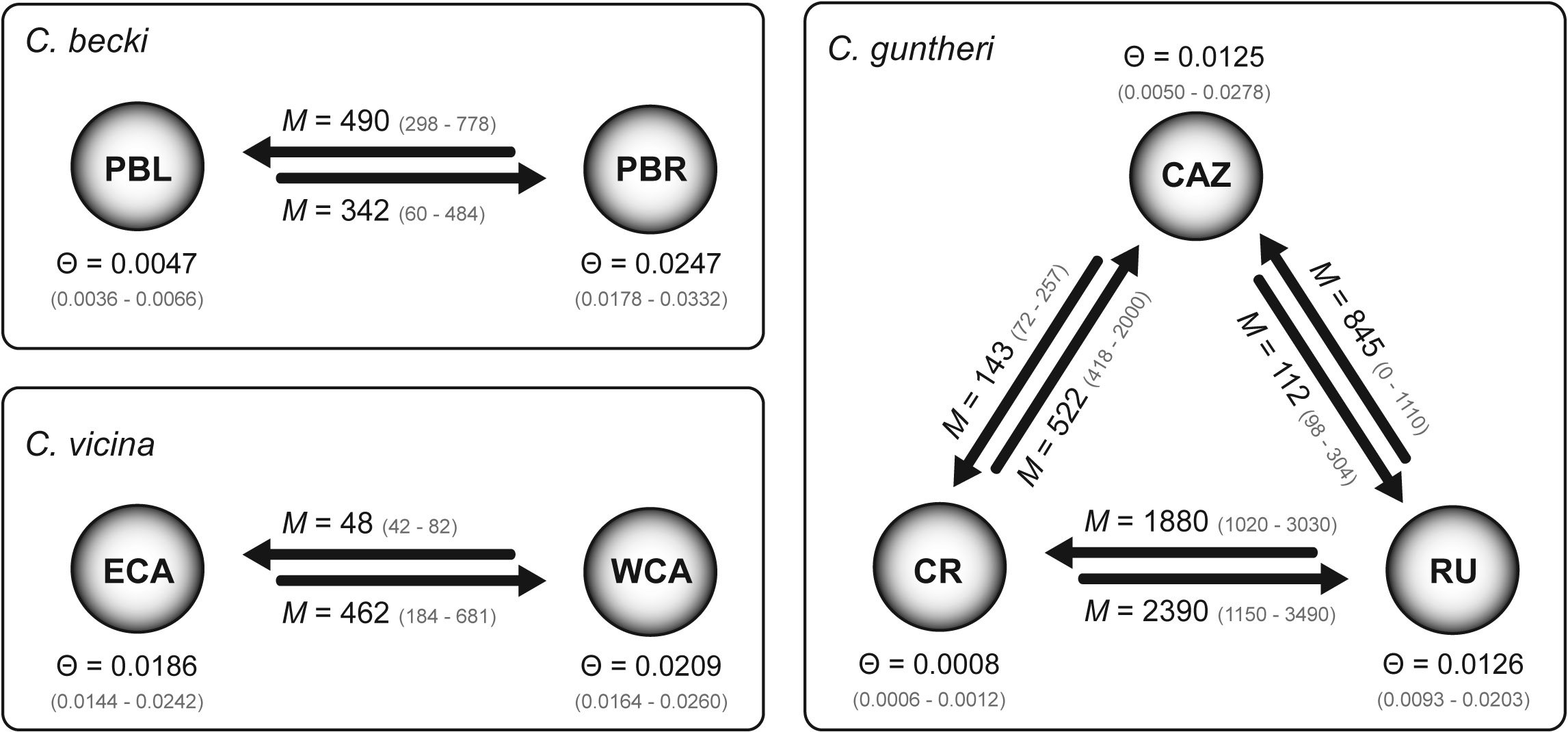


**Fig. S4.** Migration matrices estimated using MIGRATE (Beerli & Felsenstein 2001), based on multilocus DNA sequence data (mt*CR* plus *PAX1P1*), for the three tortoise species for which multiple local populations exist. Maximum likelihood point estimates of parameters included in the full two- or three-population model are given in black text, and 90% confidence intervals are in grey text in parentheses. The parameter θ = *N_e_*µ for mt*CR*, or 4*N_e_*µ for *PAX1P1*, and the parameter *M* is the mutation-scaled immigration rate, *m*/µ. Population abbreviations follow Fig. 1 of the main text.


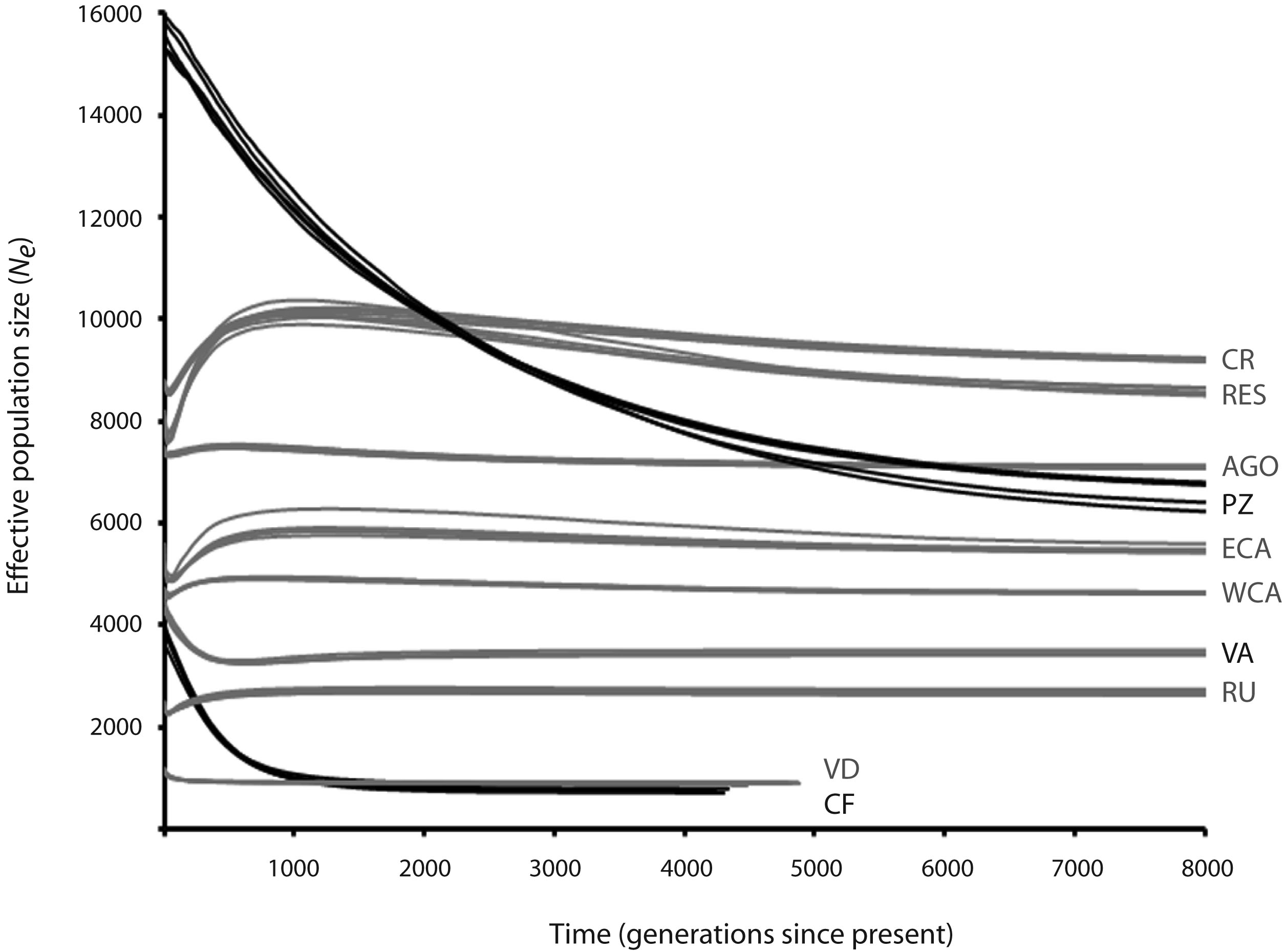


**Fig. S5.** Extended Bayesian skyline plot analysis of changes in *N*_e_ over time, jointly estimated from *PAX1P1* and mt*CR* sequences, using BEAST (Drummond & Rambaut 2007). Population abbreviations follow Fig. 1 of the main text. Curves represent the median *N*_e_-value (five replicates each). Black curves represent populations with strong evidence of growth, whereas grey curves represent those with stable size (i.e., the modal number of population size changes > 0 *vs*. = 0, respectively). Curves were cropped at 8,000 generations (200 KYA) to facilitate comparison.

**Supplementary References**

Beerli P, Felsenstein J (2001) Maximum likelihood estimation of a migration matrix and effective population sizes in *n* subpopulations by using a coalescent approach. *Proceedings of the*

*National Academy of Sciences, USA*, **98**, 4563–4568.

Do C, Waples RS, Peel D, Macbeth GM, Tillett BJ, Ovenden JR (2014) NeEstimator V2: Re-implementation of software for the estimation of contemporary effective population size (*N_e_*) from genetic data. *Molecular Ecology Resources*, **14**, 209–214.

Drummond AJ, Rambaut A (2007) BEAST: Bayesian evolutionary analysis by sampling trees. *BMC Evolutionary Biology*, **7**, 214

Evanno G, Regnaut S, Goudet J (2005) Detecting the number of clusters of individuals using the software STRUCTURE: A simulation study. *Molecular Ecology*, **14**, 2611–2620.

Excoffier L, Laval G, Schneider S (2005) Arlequin (version 3.0): An integrated software package for population genetics data analysis. *Evolutionary Bioinformatics Online*, **1**, 47–50

Fu Y-X (1997) Statistical tests of neutrality of mutations against population growth, hitchhiking and background selection. *Genetics*, **147**, 915–925.

Garrick RC, Benavides E, Russello MA *et al.* (2012) Genetic rediscovery of an ‘extinct’ Galápagos giant tortoise species. *Current Biology*, **22**, R10–R11.

Garza JC, Williamson EG (2001) Detection of reduction in population size using data from microsatellite loci. *Molecular Ecology*, **10**, 305–318.

Heled J, Drummond AJ (2008) Bayesian inference of population size history from multiple loci. *BMC Evolutionary Biology*, **8**, 289.

Kuhner MK, Yamato J, Felsenstein J (1998) Maximum likelihood estimation of population growth rates based on the coalescent. *Genetics*, **149**, 429–434.

Lessa EP, Cook JA, Patton JL (2003) Genetic footprints of demographic expansion in North America, but not Amazonia, during the late Quaternary. *Proceedings of the National Academy of Sciences, USA*, **100**, 10331–10334.

Librado P, Rozas J (2009) DnaSP v5: A software for comprehensive analysis of DNA polymorphism data. *Bioinformatics*, **25**, 1451–1452

Lynch M, Ritland K (1999) Estimation of pairwise relatedness with molecular markers. *Genetics*, **152**, 1753–1766.

Piry S, Luikart G, Cornuet J-M (1999) Bottleneck: A computer program for detecting recent reductions in the effective population size using allele frequency data. *Journal of Heredity*, **90**, 502–503.

Pritchard JK, Stephens M, Donnelly P (2000) Inference of population structure using multilocus genotype data. *Genetics*, **155**, 945–959.

Ramos-Onsins SE, Rozas J (2002) Statistical properties of new neutrality tests against population growth. *Molecular Biology and Evolution*, **19**, 2092–2100.

Rogers AR, Harpending HC (1992) Population growth makes waves in the distribution of pairwise genetic differences. *Molecular Biology and Evolution*, **9**, 552–569.

Schneider S, Excoffier L (1999) Estimation of past demographic parameters from the distribution of pairwise differences when the mutation rates vary among sites: Application to human mitochondrial DNA. *Genetics*, 152, 1079–1089.

Wang J (2007) Triadic IBD coefficients and applications to estimating pairwise relatedness. *Genetical Research*, **89**, 135–153.

Wang J (2011) COANCESTRY: a program for simulating, estimating and analysing relatedness and inbreeding coefficients. *Molecular Ecology Resources*, **11**, 141–145.
